# Genotyping of HCV RNA Reveals That 3a Is the Most Prevalent Genotype in Mardan, Pakistan

**DOI:** 10.1155/2014/606201

**Published:** 2014-02-26

**Authors:** Sajid Ali, Ayaz Ahmad, Raham Sher Khan, Sanaullah Khan, Muhammad Hamayun, Sumera Afzal Khan, Amjad Iqbal, Abid Ali Khan, Abdul Wadood, Taj Ur Rahman, Ali Hydar Baig

**Affiliations:** ^1^Department of Biotechnology, Abdul Wali Khan University Mardan, Mardan 23200, Pakistan; ^2^Center of Biotechnology & Microbiology, University of Peshawar, Peshawar 25120, Pakistan; ^3^Department of Biotechnology, KUST, Kohat 26000, Pakistan; ^4^Department of Botany, Abdul Wali Khan University Mardan, Mardan 23200, Pakistan; ^5^Department of Agriculture, Abdul Wali Khan University Mardan, Mardan 23200, Pakistan; ^6^Department of Biochemistry, Abdul Wali Khan University Mardan, Mardan 23200, Pakistan; ^7^Department of Chemistry, Abdul Wali Khan University Mardan, Mardan 23200, Pakistan

## Abstract

The clinical outcomes of patients infected with hepatitis C virus (HCV) range from acute resolving hepatitis to chronic liver diseases such as liver cirrhosis or hepatocellular carcinoma. Identification of the infecting virus genotype is indispensable for the exploration of many aspects of HCV infection, including epidemiology, pathogenesis, and response to antiviral therapy. 1419 individuals were screened for anti-HCV in this study, of which 166 (11.7%) were found reactive by ICT (Immunochromatographic test). These 166 anti-HCV positive and 26 normal individuals were further analyzed. RNA was extracted from serum and reverse-transcribed to cDNA and the core region of HCV genome was targeted and amplified by multiplex PCR. HCV RNA was detected in 121 individuals, of which 87 were male and 34 were female. Genotype 3a was the most prevalent among all the genotypes observed followed by 3b. Genotypes 1a, 2a, and 2b were found in 10.89%, 13.22%, and 6.61% patients, respectively. 25.41% of the HCV RNA positive samples were not typed. 6.05% of patients were found having mixed genotypes. These findings will not only help the physicians to prescribe more appropriate treatment for the HCV infection but will also draw the attention of health-related policy makers to devise strategies to curb the disease more effectively.

## 1. Introduction

Hepatitis C virus (HCV) is the most frequent cause of chronic viral hepatitis worldwide. In the recent years, infection with HCV has emerged as one of the most common causes of acute and chronic liver diseases all over the world [1]. HCV is a member of the *Flaviviridae *family that bears approximately 10 kb long positive sense single-stranded RNA (ssRNA) genome. Since anti-HCV testing alone cannot differentiate between acute, chronic, or resolved infection, a supplementary test must also be carried out, involving measurement of anti-HCV immunoglobulin G activity index [2] or antibody reactivities to specific HCV structural and nonstructural proteins [3], to confirm a positive anti-HCV result [1]. HCV is known to have high rate of genetic heterogeneity [4]. This has allowed HCV strains to be classified into a number of genetically distinct groups, known as genotypes, subtypes, isolates, and quasispecies [5]. The genetic variability among HCV strains is 65.8%–68.7% nucleotide sequence identities of full-length sequences for types, 76.9%–80.1% nucleotide sequence identities of full-length sequences for subtypes, and 90.8%–99% nucleotide sequence identities of full-length sequences for isolates and quasispecies [6]. Six major genotypes, that is, 1 through 6 and more than 50 subtypes, have been identified so far. These genotypes differ by 31 to 34% in their nucleotide sequences whereas the subtypes differ by 20 to 23% in their full-length genomic sequences. This extensive genetic heterogeneity, as well as the tendency for mutation, has hindered vaccine development against this virus [5]. As patients infected with different genotypes respond differently to antiviral drug therapy, identification of the infecting genotype is inevitable to guide the correct dose and duration of current combination therapy (pegylated alpha interferon plus ribavirin) [7].

Hepatitis C is also common in Pakistan but accurate epidemiological information is quite limited. In the outer edges of the cities and in remote areas, unqualified medical and dental practitioners, lady health visitors, midwives, and barbers often use unsterilized instruments which are major potential sources of spreading HCV infection in the urban and rural population of Pakistan [8]. Although the exact ratio of this chronic disease is not known, various studies have shown that 3–7% of population of Pakistan is infected [9]. An earlier study reported that 8.9% of population is infected with HCV in Mardan [10], which is the second largest city in Khyber Pakhtunkhwa province of Pakistan. It is significantly prominent to inspect the degree and distribution of HCV genotypes in district Mardan, Khyber Pakhtunkhwa, where detection and genotype determination preceding therapy are sporadic. Therefore, appropriate information to make individual treatment is required in order to maximize the chance of successful treatment outcome for each individual patient, rendering HCV genotyping assays important and useful tools to optimize treatment type, duration, and dose. This study was designed to determine the active HCV RNA infection as well as to know about the HCV genotypes circulating in the study area.

## 2. Materials and Method

All the blood samples were collected from different areas (hospitals and clinical laboratories) of Mardan. A 5 cc of blood was taken in a Vacutainer tube and serum was isolated or whole blood was stored at −80°C for further analysis at the Department of Biotechnology, KUST. While collecting blood samples, a proforma was filled to collect medical/clinical information from all the individuals and obtain their consent. Patient's history, liver function tests (LFTs), jaundice, blood transfusion, and other different tests such as HBs Ag and anti-HCV, if carried out, were noted. This study was conducted with the approval of ethics committee of Kohat University of Science and Technology (KUST) Kohat, Pakistan.

### 2.1. Immunochromatographic Test (ICT)

Initially all the samples were screened for HCV antibodies using ICT device kit (Accurate Diagnostic, Canada), as described according to the manufacturer instructions.

### 2.2. RNA Extraction and cDNA Synthesis (for 5′UTR Detection)

RNA was extracted from 300 *μ*L of blood sample by using RNA extraction kit (RNA purification kit, Ultrascript, Anagen Technologies, Inc., USA). cDNA was synthesized from the 5′UTR region of extracted RNA, using primers.

### 2.3. Regular PCR (for 5′UTR Detection)

cDNA synthesized was then amplified in next round of regular PCR using a sense and an antisense primer specific for 5′UTR [11]. cDNA was used from the previous round and was run in thermocycler. The cycling conditions for regular PCR were consisting of 25 cycles in three steps I, II, and III.

### 2.4. Nested PCR (for HCV 5′UTR Detection)

After regular PCR, nested PCR was carried out using the next pair of primers internal to the first one [11]. A mixture was prepared in the same way as for regular PCR except the primers. The PCR cycling conditions were the same as for regular PCR.

### 2.5. Electrophoresis

12 *μ*L of the amplified cDNA was resolved on 2% agarose gel. cDNA bands (230 bp) were identified by comparing with a DNA ladder marker of 100 bp (Fermentas, USA) and visualized under UV illumination using gel documentation system.

### 2.6. HCV Genotyping

#### 2.6.1. cDNA Synthesis for Core Region

cDNA was synthesized from the core region of extracted RNA with specific primers [11]. The reaction was carried out in thermocycler (Techne Inc., USA) using the same program as for 5′UTR.

#### 2.6.2. HCV 1st Round PCR for Genotyping

Two primers (one reverse and one forward) [11] specific for core region of HCV were used to amplify cDNA in a thermocycler (Techne Inc., USA) with *Taq *DNA polymerase (Fermentas, USA).

#### 2.6.3. HCV Genotype-Specific PCR

Genotyping with type-specific primers [11] for the core region of HCV genome was performed for the nine most common subtypes and types of HCV (1a, 1b, 2a, 2b, 3a, 3b, 4, 5a, and 6a). For the discrimination of different products of the HCV genotypes amplified, the type-specific PCR mix was divided into mix A and mix B. For mix B all the reagents were the same except the antisense primers. The HCV genotype-specific PCR/multiplex PCR was performed using the same program as for 1st round of PCR.

#### 2.6.4. Electrophoresis

PCR products were resolved on 2% agarose gel, prepared in 0.5X TBE buffer, and visualized under ultraviolet light. HCV genotypes were determined by comparing the amplified product (cDNA bands) of a specific genotype with 100 bp DNA ladder marker (Fermentas, USA).

## 3. Results

**Figure 1:**
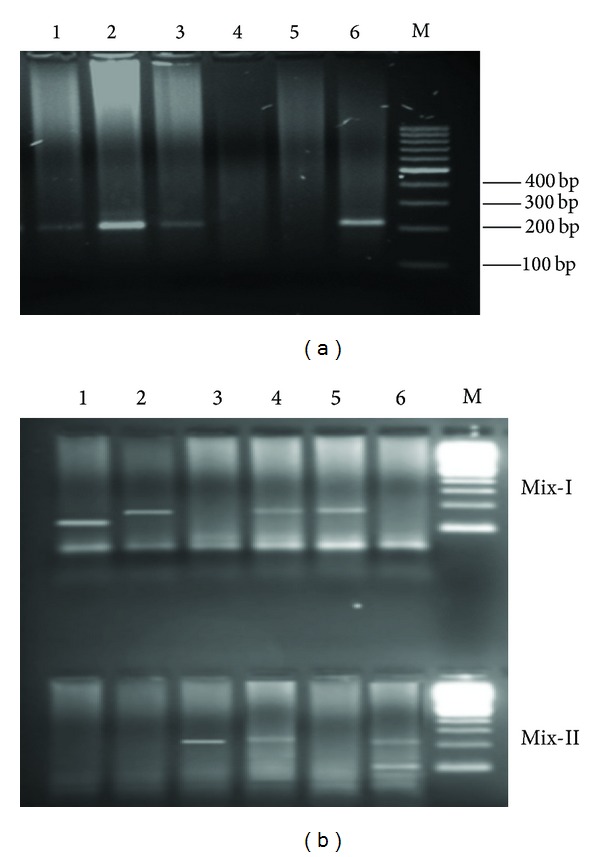
(a) Screening of samples for HCV RNA by amplifying 5′UTR. Lanes 1, 2, and 3: positive samples (232 bp band of 5′UTR), Lane 4: negative sample, Lane 5: negative control, Lane 6: positive control, and Lane M: 100 bp DNA ladder. (b) Amplified products of different HCV genotypes. Lane 1 = 139 bp (HCV genotype 2a), Lane 2 = 176 bp HCV genotype 3b, Lane 3 = 232 bp (HCV genotype 3a), Lane 4 = 176 and 232 bp (HCV genotypes 3b and 3a), Lane 5 = 176 bp HCV genotype 3b, Lane 6 = 232 and 99 bp (HCV genotypes 3a and 4), and Lane M = 100 bp DNA ladder.

A total of 1419 subjects were screened for anti-HCV, of which 166 were found positive for HCV antibodies, comprising 11.7% of the screened population (Figure 1 and Table 1). Of the total screened individuals, 192 were selected for further study, such that 166 were anti-HCV positive and 26 were from anti-HCV negative (normal individuals), as control, with no apparent history or symptoms of hepatitis C. 103 males and 63 females were anti-HCV positive whereas in general population 17 males and 9 females were anti-HCV negative (Table 1).

All subjects were analyzed with PCR for HCV RNA detection. Of the total 192 individuals, 121 were found to be HCV RNA positive while 71 were negative. Of these 121 HCV RNA positive individuals, 87 were males and 34 were females. HCV RNA was not detected in 16 males and 29 females which were anti-HCV positive. In all 26 anti-HCV negative individuals (general population), HCV RNA was not observed after amplification with nested PCR which indicates true negative controls.

**Figure 2:**
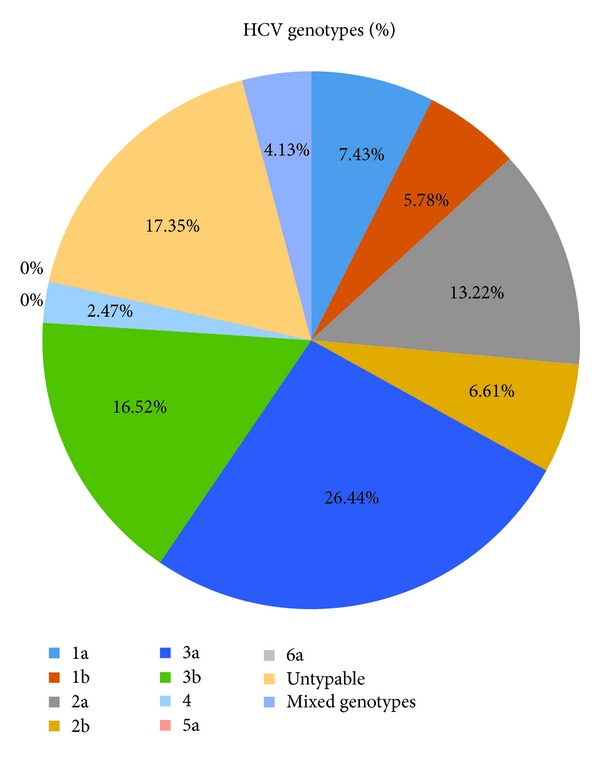
Percentage of HCV genotypes.

The prevalence of different genotypes in the subject population was analyzed by type-specific PCR targeting the core region of HCV genome. HCV genotype 3a was detected in 26.44% of the anti-HCV positive individuals, the most prevalent genotype in the studied area. HCV genotype 3b was observed in 16.52% followed by genotype 2a, 13.22%, and genotype 1a was 7.43%. HCV genotypes 5a and 6a were not detected in any of the HCV RNA positive patients. 17.35% of samples were such that where no genotype was observed and were declared as untypable by the method used. Mixed infection of HCV genotypes was found in 4.13% samples (Figure 2).

The chances of HCV infection in males are relatively higher than females because of more frequent visits of males to high risk areas like barber, and so forth, in this region. The genotype 3a was found to infect males more frequently followed by 3b and 2a, respectively, whereas genotypes 2b and 1a were found to be the major causes of infections in females. Data was analyzed using Roy's Largest Root for correlation of HCV genotype to gender and was found nonsignificant (RLR = 0.834, *P* > 0.05 NS) (Table 2).

## 4. Discussion

Hepatitis C virus (HCV) has variable clinical outcomes in different infected patients ranging from acute resolving hepatitis to chronic liver diseases including liver cirrhosis and Hepatocellular Carcinoma (HCC) [12]. Identification of the infecting virus genotype is important for the exploration of many aspects of HCV infection, including epidemiology, pathogenesis, and response to antiviral therapy [13, 14]. A suitable and reliable HCV genotyping method is inevitable for large-scale epidemiological and experimental studies [11]. A number of laboratory procedures aimed at identifying the HCV genotypes have been described [15]. HCV genotype determination in full-length genomic sequence analysis followed by phylogenetic analysis is still the golden standard. Though this system is expensive and time consuming and cannot be adapted to clinical studies or extensive standard use [15]. PCR has been broadly used for genotyping [13], which is based upon the amplification of virus sequences in clinical specimens, using type-specific primers that specifically amplify different genotypes [11, 16]. Information obtained from various parts of the world has focused on the increasing implication of HCV genotyping and stressed the need of easy, reliable, cost effective, and fast techniques for mass screening.

The precise and sensitive measurement of HCV RNA is important for medical management of infected patients and to know about the biology of HCV and HCV infection in research [17]. The clinical causes, risk factors, and severity of HCV infections and HCV genotypes are still poorly defined in district Mardan, like other districts of Khyber Pakhtunkhwa. Antibodies against HCV were observed in 11.70% of the studied population, which shows high prevalence in comparison to 4.57% reported by Muhammad and Jan in district Buner [18].

Our data showed that genotype 3a (26.44%) followed by genotype 3b (16.52%) is prevalent in district Mardan and the study of Idrees and Riazuddin [19], where genotypes 3a and 3b were dominant among the studied population [19]. Also in another study by Idrees and Riazuddin, HCV genotype 3a was found predominant in general population [19]. Regarding occurrence of HCV genotypes in other countries like Argentina, genotype 2 is more prevalent [20]. Similarly, Alfonso et al. (2001) observed genotype 1 in 61.4% and genotype 3 in 23.7% infected patients in Latin American region [21]. In a recent review we reported that genotype 3a accounts for 68.94% of HCV infections in Punjab, 76.88% in Sindh, 58% in KPK, and 60.71% in Balochistan provinces of Pakistan [22].

Analysis of epidemiological data showed marked differences between patients with single infections and those with apparently mixed infections illustrated by the type-specific PCR [13]. Mixed infection was observed in our study in 4.13% of samples (Figure 2). The mixed genotypes may be due to infection by different HCV types by repeated exposure to HCV [23]. In addition, 17.35% of the individuals which were confirmed by nested PCR for HCV RNA were not typed by type-specific PCR. That is not in agreement with our previous findings that showed 20.16% of the HCV positive individuals in KPK were untypable [22].

HCV is known as silent killer because in majority of the cases no proper signs or symptoms are visible in the early stages of infection and when symptoms appear then the treatment is difficult. Secondly, in most of the developing countries diagnosis is not proper due to lack of facilities. The people are screened for anti-HCV through strips or by anti-HCV ELISA test for HCV infection but these methods are more erroneous and lack sensitivity [24]. Therefore, molecular detection of HCV by PCR based methods is inevitable due to higher levels of sensitivity and specificity than the serological methods. HCV infection is a typical example of diseases in which direct detection of the virus is essential for a correct diagnosis. In comparison to other existing in vitro assays, RT-PCR has extra prospective for its diagnosis as it offers an ultimate detection of HCV [25].

The present study shows that the distribution of HCV genotype 3 in Mardan is similar to that in other areas of Pakistan. HCV types 2 and 3 are prevalent in this area which can better respond to interferon therapy but types 1 and 4 were also circulating which need longer treatment. Proper epidemiological studies and treatment strategies should be initiated in this area. Appropriate preventive measures should be also taken into consideration to control the spread of this dreadful disease.

## Figures and Tables

**Table 1:** Screening general population of Mardan for anti-HCV and HCV RNA.

	Male	Female	Total (male + female)
Total subjects	757	662	1419
Anti-HCV +Ve	103	63	166
HCV RNA +Ve	87	34	121

**Table 2:** Gender-wise distribution of HCV genotypes.

Genotypes	Male	Female
1a	6	3
1b	5	2
2a	11	5
2b	2	6
3a	25	7
3b	16	4
4	2	1
Mixed genotypes	4	1
Untypable	17	4

Roy's Largest Root = 0.834, *P* > 0.05 NS.
